# Smallanthus sonchifolius leaf attenuates neuroinflammation

**DOI:** 10.20463/jenb.2018.0014

**Published:** 2018-06-30

**Authors:** Suji Baek, Nan Hee Choi, Kang-Pa Lee, Hyunjhung Jhun, Jisu Kim

**Affiliations:** 1 Department of Medical Science, School of Medicine, Konkuk University, Seoul Republic of Korea; 2 Department of Biotechnology, College of Engineering, Daegu University, Gyeongsan Republic of Korea; 3 Research Group of Nutraceuticals for Metabolic Syndrome, Korea Food Research Institute, Seongnam Republic of Korea; 4 Physical Activity & Performance Institute, Konkuk University, Seoul Republic of Korea

**Keywords:** Alzheimer’s disease, anti-inflammation, lipopolysaccharide, microglia, yacon leaf extract

## Abstract

**[Purpose]:**

Yacon, *Smallanthus sonchifolius*, has anti-hypertensive, anti-inflammatory, and anti-cancer potential. However, its neuroprotective and anti-neuroinflammatory effects are unknown. Moreover, activation of microglia has been considered a mechanism in the development of Alzheimer’s disease. Therefore, the aim of this study was to determine the neuroprotective effects of an ethanolic yacon leaf extract (YLE) on lipopolysaccharide (LPS)-induced neuroinflammation *in vitro* and *in vivo*.

**[Methods]:**

The viability of microglial BV2 cells was tested with 2,3-bis[2-methyloxy-4-nitro-5-sulfophenyl]-2H-tetrazolim-5-carboxanilide. The production of nitric oxide (NO) was determined by the Griess reagent. mRNA expression and protein levels of inflammatory mediators were evaluated by the real-time polymerase chain reaction and immunohistochemistry, respectively. In addition, we performed histological analysis in mice treated with an intraperitoneal injection of LPS (250 μg/kg).

**[Results]:**

Our results showed that treatment with YLE significantly reduced NO production in LPS-stimulated BV2 cells. YLE also decreased mRNA levels of the inflammatory factors tumor necrosis factor alpha, inducible nitric oxide synthase, cyclooxygenase-2, and interleukin-1 beta. In vivo, YLE (40 mg/kg daily for seven days) significantly diminished LPS-induced tissue damage in the dentate gyrus and cornu amonis regions of the hippocampus by regulating the levels of inflammatory factors.

**[Conclusion]:**

Our findings support the protective effects of YLE against the development of neurodegeneration.

## INTRODUCTION

Alzheimer’s disease (AD) is a neurodegenerative disorder characterized by a progressive loss of memory^1^. Although the precise causes of AD are etiologically complex, the dominant theory is that cell death is the main cause of β-amyloid (Aβ) accumulation in the brain^2,3^. Furthermore, inflammation can eventually lead to progressive neuronal degenerative diseases through Aβ deposition which influences the destruction of healthy tissue.

Abnormal activity of microglia, the resident macrophage cell in the central nervous system, can lead to the accumulation of Aβ^4^. Consequently, microglial activation contributes to the pathogenesis of neurodegenerative disorders^5^. In particular, up-regulation of inflammatory factors such as inducible nitric oxide synthase (iNOS), cyclooxygenase (COX)-2, interleukin-1 beta (IL-1β), and tumor necrosis factor alpha (TNF-α) have been observed in inflamed brain tissue^6^. Because activated microglia accumulate Aβ induced by lipopolysaccharide (LPS), regulating inflammatory factors is one of the strategies employed in experimental trials for AD^7^.

Some functional foods contain active ingredients that reduce the risk of neuronal dysfunction^8^. Yacon (*Smallanthu sonchifolius*), as a functional food, has been shown to prevent disease and promote health. Based on several preclinical and clinical trials, yacon ingestion can be used effectively as a dietary supplement to prevent colon cancer and to promote health through controlling the immune response, glucose homeostasis, and lipid metabolism^9^. However, its neuroprotective effects have not been evaluated thus far.

In this study, we investigated the anti-inflammatory activity of yacon leaf extract (YLE) on LPS-stimulated mouse microglia (BV2) cells in vitro. Furthermore, mRNA levels of the inflammatory factors iNOS, COX-2, IL-1β, and TNF-α were measured. In addition, we demonstrated the neuroprotective effect of YLE in an LPS-induced mouse model of AD. Our results show the biological activity of YLE against LPS-induced neurodegeneration, offering a potential functional food for neuroprotection.

## METHODS

### Chemicals

Cell culture materials were purchased from Sigma-Aldrich (St. Louis, MO, USA). iNOS, COX-2, and TNF-α antibodies were obtained from Cell Signaling Technology (Danvers, MA, USA). All other reagents were purchased from Sigma-Aldrich.

### Preparation of ethanolic YLE

Yacon leaves were extracted with ethanol as reported previously^10^. Briefly, yacon leaves were obtained from Dea-Jea Farm (Andong, Korea). Two hundred grams of the plant were blended and the crude powder boiled in 1 L of sterile deionized water at 100°C for 3 h. The remaining debris was precipitated with 700 mL of absolute ethanol at 4°C for 3 days. The aqueous extracts were concentrated and evaporated at 60°C under vacuum conditions. The decocted solution was dissolved in 50 mL of sterile deionized water. The aqueous extract was lyophilized at −60°C.

### Cell culture and cell viability

Cell culture was performed, and cell viability was assessed as in our previous report^11^. BV2 cells were obtained from the Korean Cell Line Bank (Seoul, Korea) and grown in Dulbecco’s modified Eagle’s medium containing 10% fetal bovine serum and 1% penicillin-streptomycin at 37°C under 5% CO2. BV2 cells were seeded at a density of 5 × 104 cells/mL in 96-well plates. Cells were treated with YLE (10, 25, or 50 μg/mL) for 48 h and then incubated with 2,3-bis[2-methyloxy-4-nitro-5-sulfophenyl]-2H-tetrazolim-5-carboxanilide reagent for 2 h at 37°C in the dark. Cell viability was calculated as the percentage of surviving cells by measuring absorbance at 450 nm.

### Analysis of nitric oxide (NO) production

NO production by LPS-stimulated BV2 cells was determined by the Greiss reagent as described previously [11]. Briefly, BV2 cells (5 × 104 cells/well) were treated with different concentrations of YLE (10 to 50 μg/mL) in the absence or presence of LPS in Dulbecco’s modified Eagle’s medium for 48 h. NO in 100 μL of culture media was determined as the nitrate concentration by measuring absorbance at 520 nm.

### Real-time polymerase chain reaction (PCR)

mRNA expression levels of the inflammatory factors iNOS, COX-2, IL-1β, and TNF-α were measured by real time-PCR as described previously^11^. Briefly, total RNA was isolated from cell lysates using TRIzol reagent following the manufacturer’s protocol. First-strand cDNA was synthesized using SuperScript II Reverse Transcriptase following the manufacturer’s protocol . cDNA was amplified by the real-time PCR and a thermocycler under the following conditions: initial denaturation for 10 min at 95°C, followed by 40 cycles of denaturation (30 s, 95°C), annealing (30 s, 60°C), and extension (30 s, 72°C). Relative gene expression levels were determined by calculating the Δcycle threshold value (ΔCt), normalizing the average Ct value to the control GAPDH, and then calculating 2^-ΔΔCt^.

### Animal care and in vivo studies

ICR male mice (20–23 g) were purchased from Orient Bio (Seongnam, Korea). All mice were kept in a controlled environment (24 ± 2°C; humidity, 40 ± 2%; 12 h light/dark cycle). All experiments and animal care were conducted in accordance with the institutional guidelines of Dongguk University (IACUC-2014-005). Animals (6 weeks old; n = 30) were divided randomly into three groups. The untreated group (control) received a saline solution. The LPS-induced brain damage group (LPS) received a single intraperitoneal injection of 250 μg/kg LPS . The treatment group received YLE (40 mg/kg/day orally for seven days) in normal saline, and LPS after 1 h.

### Histochemistry and immunohistochemistry

Histochemistry and immunohistochemistry were performed as described previously^12^. Animals were sacrificed after seven days and the brain was dissected. The brain was washed in ice-cold phosphate-buffered saline and fixed in 2% formalin. Segments from each half were embedded in paraffin and serially sectioned into 5-μm slices. The prepared sections were cleared with xylene and hydrated with serial concentrations of ethanol (70, 80, and 90%). Sections were stained with hematoxylin and eosin or cresyl violet. Some sections were incubated overnight at 4°C with primary antibodies for iNOS, COX-2, and TNF-α.

### Statistical analyses

Results are expressed as means ± standard error of at least three independent experiments. Differences between two groups were determined using Student’s *t*-test. Multiple comparisons were performed by one-way analysis of variance followed by Tukey’s post-hoc test (GraphPad Prism ver. 4.00 for Windows, GraphPad, La Jolla, CA, USA). *p*-values < 0.05 were considered statistically significant.

## RESULTS

### YLE reduces NO production in LPS-stimulated BV2 cells

**Fig. 1.**
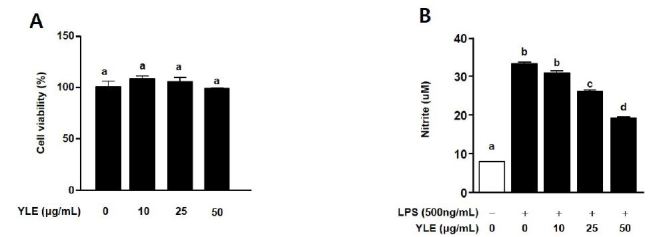
Effects of ethanolic yacon leaf extract (YLE) on lipopolysaccharide (LPS)-induced BV2 microglial cell viability and the production of nitric oxide (NO). (A) BV2 cells were treated with YLE (10, 25, and 50 μg/mL) for 48 h and cell viability was examined using 2,3-bis [2-methyloxy-4-nitro-5-sulfophenyl]-2H-tetrazolim-5-carboxanilide (n = 4). Cell viability in the quiescent state is expressed as 100%. (B) Cells were co-treated with YLE and LPS (500 ng/mL) for 48 h. The production of NO in BV2 cells was determined using the Griess reagent (n = 6). Data are expressed as means ± SE. Values with the same letter are not significantly dfiferent, as determined byT ukey’s multiple range test (p < 0.05).

Cell viability analyses showed no change following treatment with YLE at concentrations up to 50 μg/mL (Fig. 1A). As shown in Figure 1B, the NO level in LPS-stimulated cells was 33.6 ± 0.4 μM (*p* = 0.0001 vs. unstimulated cells). LPS-induced NO production decreased to 2.3 ± 0.2 (*p* = 0.0347), 7.8 ± 0.5 (*p* = 0.0005), and 14.1 ± 0.1 μM (*p* = 0.0039) at YLE concentrations of 10, 25, and 50 μg/mL, respectively.

### YLE reduces the expression of inflammatory signals in LPS-stimulated BV2 cells

**Fig. 2.**
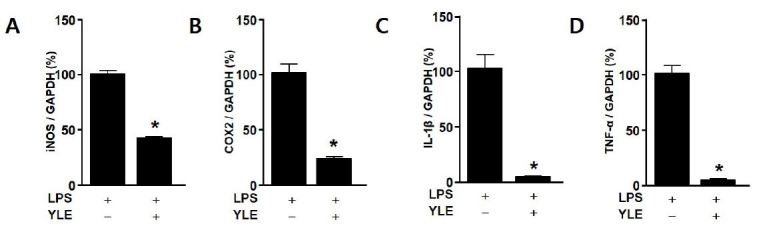
Effects of ethanolic yacon leaf extract (YLE) on lipopolysaccharide (LPS)-induced mRNA expression of proinflammatory cytokines in BV2 cells. BV2 cells (2 × 105 cells) were seeded in 100 mm dishes for 24. Thhe cells were then incubated in the absence or presence of LPS (500 ng/mL) anYd LE (50 μg/mL) for 24 h. mRNA levels of inducible nitric oxide synthase (iNOS), cyclooxygensae (COX)-2, interleukin (IL)-1β, and tumor necrosis factor (TNF)-α were assessed by the real-time polymerase chain reaction. (A–D) Relative mRNA expression compared with the LPS-treated group (100%). Data are expressed as means ± SE (n = 3). *Significant difference compared with LPS-only treatment (p < 0.05).

Figure 2 shows that 50 μg/mL YLE decreased LPS-induced iNOS, COX-2, IL-1β, and TNF-α mRNA expression by 57.5 ± 3.4 (*p* = 0.0001), 77.6 ± 5.4 (*p* = 0.0013), 97.4 ± 11.1 (*p* = 0.0026), and 95.7 ± 6.1% (*p* = 0.0003 ), respectively.

### Histological analysis of the hippocampus from LPS-treated mice

**Fig. 3.**
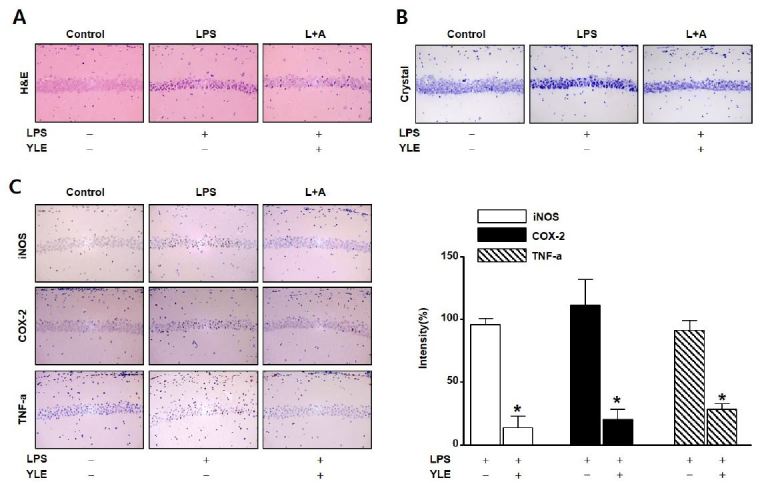
Histological analysis of hippocampus following lipopolysaccharide (LPS)-induced neuroinflammation. LPS (250 μg/kg) was injected intraperitoneally into male ICR mice. Brains were sectioned after 7 days. pHpiocampus morphology was analyzed using hematoxylin and eosinA () and cresyl violet (B ) staining. (C) The inflammatory factors inducible nitric oxide synthase (iNOS), cyclooxygenase (COX)-2, and tumor necrosis factor (TNF)-α were detected using immunohistochemical staining in cross-sections. Bar graphs quantitate the intensity of the brown color representing the expression of iNOS, COX-2, and TNF-α. Protein expression in the LPS alone group is considered 100%. Data are expressed as means ± SE (n = 3). *Significant difference compared with the LPS alone group (p < 0.05).

A previous study demonstrated that intraperitoneal injection of LPS induced mouse brain damage^13^. Figure 3A and B shows the morphology of the dentate gyrus (DG) and cornu amonis (CA) regions in the hippocampus from LPS-treated mice. Oral administration of YLE (40 mg/kg/day) significantly reduced thin layer formation following LPS-induced brain damage. Analysis of inflammatory factors and pro-inflammatory cytokines showed that YLE significantly reduced inflammatory signals in the LPS-stimulated CA1 region. Specifically, YLE reduced the levels of LPS-stimulated iNOS, COX-2, and TNF-α by 81.7 ± 8 (*p* = 0.0002), 89.9 ± 12.1 (*p* = 0.0002), and 62.8 ± 3.4% (*p* = 0.0003), respectively (FIG. 3C ).

## DISCUSSION

In the present study, we focused on YLE as a potential functional food for decreasing inflammation. The results demonstrate that YLE treatment in LPS-induced neuroinflammation regulated the expression of inflammatory factors both in vitro and in vivo. These results also indicated that YLE treatment attenuated damage to the DG and CA regions in brains from LPS-treated mice.

Excessive activation of microglia is implicated in neuronal disorders including neuronal cell death and neuroinflammation. LPS stimulation causes excessive secretion of pro-cytokines in the brain^14^. Up-regulating the inflammatory pathway is associated with the accumulation and deposition of Aβ^15^,^16^. To our knowledge, we provide one of the first validations that treatment with YLE significantly reduces neuronal damage from LPS-stimulated microglia.

Although increased NO in microglia is important for defense against pathogens, excessive production of NO by activated microglia has crucial involvement in neuroinflammation and brain damage^17^. LPS exacerbates microglia activities, such as the induction of NO, which can lead to neuronal cell damage^18^. Inflammation plays an important role in neurodegenerative disorders such as AD or dementia. Our results show that YLE significantly reduces NO in LPS-stimulated BV2 cells. Therefore, we suggest that YLE is a potential functional food for neuroprotection.

It is important that the in vivo dosage of functional foods be associated with a lack of toxicity because of the possibility for long-term use. Despite the short-term yacon intake involved in the current study, our immunohistochemistry data showed that YLE inhibited neuroinflammation. Furthermore, Oliveira et al. reported that a high level of yacon intake (1000 mg/kg for 90 days) was non-toxic^19^. Therefore, our results suggest that YLE is a functional food candidate that can be taken for a long time to decrease neuroinflammation.

Diverse studies have reported that iNOS, COX-2, IL- 1β, and TNF-α are important factors in LPS-induced inflammation^20^. Especially, activation of the microglia signaling pathway includes components such as TNF-α and pro-inflammatory cytokines^21^. Baker et al. supported the idea that NF-κB induced transcription in response to the severe inflammation after LPS administration^22^. Furthermore, LPS exacerbated microglia activities such as the induction of transcription factors and the secretion of pro-cytokines, which can lead to the accumulation of Aβ^23^. Our results showed that YLE significantly reduced the expression of pro-cytokines. Therefore, YLE is a promising candidate for developing a therapeutic agent against acute and chronic neuroinflammation .

Our results showed that treatment with YLE significantly reduced LPS-induced inflammation of mouse brain. Furthermore, diverse studies have shown that the various metabolic control actions of yacon, such as antioxidant, anti-obesity, and glucose control, are related to exercise performance . Therefore, we suggest that YLE is a potential functional food for improving athletic performance.

In conclusion, our findings suggest that YLE protects against brain damage by regulating excessive inflammation. Although we provide useful information for clinical approaches, the limited in vitro and in vivo outcomes of the present study necessitate further research to clarify the effects of YLE on athletic endurance performance.
